# Metagenomic Next-Generation Sequencing vs. Traditional Pathogen Detection in the Diagnosis of Infection After Allogeneic Hematopoietic Stem Cell Transplantation in Children

**DOI:** 10.3389/fmicb.2022.868160

**Published:** 2022-04-18

**Authors:** Yuhua Qu, Wenjiao Ding, Sha Liu, Xiaojing Wang, Pengfei Wang, Haiyan Liu, Han Xia, Yong Chen, Hua Jiang

**Affiliations:** ^1^Department of Hematology and Oncology, Guangzhou Women and Children’s Medical Center, Guangzhou, China; ^2^Department of Scientific Affairs, Hugobiotech Co., Ltd., Beijing, China; ^3^Department of Scientific Affairs, BGI PathoGenesis Pharmaceutical Technology Co., Ltd., Shenzhen, China

**Keywords:** metagenomic next-generation sequencing, allogeneic hematopoietic stem cell transplantation, pulmonary infection, central nervous system infections, pediatric

## Abstract

Infection is a severe complication of allo-HSCT in children, however, the accurate detection of the infection is hard. In this study, we traced the records of 101 pediatric recipients with allo-HSCT to investigate the pathogens of infection, and collected 54 bronchoalveolar lavage fluid, 32 blood, and 15 cerebrospinal fluid samples. In these samples, 87 was with post-transplant infection and 14 without infection. Using the metagenomic next-generation sequencing (mNGS) and traditional pathogen detection, we compared their sensitivity and specificity to detect pathogens of infection. Our results showed that mNGS was more sensitive (89.7%) than conventional pathogen detection (21.8%), with a difference of 67.9% (*P* < 0.001), However, mNGS was less specific (78.5%) than traditional methods (92.9%), with a difference of 14.4% (*P* = 0.596). The sensitivity of mNGS for diagnosing pulmonary infections, bloodstream infections or viremia, and CNS infections post-transplant were 91.7, 85.7, and 90.9%, respectively. In contrast, the sensitivity of conventional testing for diagnosing pulmonary infections, bloodstream infections or viremia, and CNS infections post-transplant were 22.9, 21.4, and 18.2%, respectively. There were significant differences in the sensitivity of mNGS and conventional testing in BALF, blood, and CSF samples, with *P* values of 0.000, 0.000, and 0.002 respectively. Among the patients with pulmonary infection, 11 pathogens were both identified by mNGS and conventional testing, and 33 by mNGS only. The percentage with the mNGS-positive result was 44/48 (91.7%), including viruses (*n* = 12), bacteria (*n* = 17), fungi (*n* = 9) and mixed infections (*n* = 6). Among the patients diagnosed with fungal pneumonia (*n* = 9), the most prevalent pathogenic fungi were *Pneumocystis jiroveci* (*n* = 6), which were also detected in 4 patients with mixed infectious pneumonia. In the 28 blood specimens of patients with bloodstream infections or viremia, five patients were positive by both mNGS and conventional testing, 19 were positive by mNGS, and 1 was positive by traditional testing only. The percentage with the mNGS-positive results was 24/28 (85.7%), including viruses (*n* = 12), bacteria (*n* = 4), fungi (*n* = 3), and mixed infections (*n* = 5). Of the 15 CSF specimens enrolled, 11 patients were eventually diagnosed with CNS infections. Ten pathogens were identified by mNGS in the 11 patients, including viruses (*n* = 8), bacteria (*n* = 1), and fungi (*n* = 1). These results suggest that mNGS is more sensitive than conventional pathogen detection for diagnosing infections post HSCT in children which may help the clinic diagnosis. *Pneumocystis jiroveci* was the most frequent pathogen of pulmonary infections post-transplant, while viruses were the most common pathogens of CNS infections in allo-HSCT recipients.

## Introduction

Allogeneic hematopoietic stem cell transplantation (allo-HSCT) is a curative option for a wide range of disorders in children, such as hematological malignancies and some non-malignant diseases, including abnormal hemoglobinopathy bone marrow debilitating diseases, inherited metabolic disorders and primary immunodeficiency diseases, etc. Immune function of patients will be reduced due to severely lymphopenic, use of immunosuppressant and graft vs. host disease (GVHD) after transplantation, and therefore prone to a more severe course of infections (Sahin et al., 2016). Infections in bloodstream, pulmonary, and central nervous system (CNS) are severe complications with high mortality in allo-HSCT recipients (Sahin et al., 2016; Rosello et al., 2017; Young et al., 2017; Ford et al., 2020; Liu et al., 2021). The incidence of various infections varied greatly after allo-HSCT. The pathogens of post-transplantation infections also varied among different reports (Maschke et al., 1999; Roemer et al., 2001; Kang et al., 2015; Young et al., 2017; Zhang et al., 2017; Ford et al., 2020). These discrepancies are partly due to a variety of testing methods. Current conventional testing, such as microbial culture, smear microscopy, histopathology, real-time quantitative polymerase chain reaction (RT-PCR), and multiplex PCR, remains commonly used to identify pathogens in clinical settings. However, there are some limitations for the above mentioned methods, for example, the positive rate for microbial culture is quite low because of antibiotic application history (Maschke et al., 1999). Secondly, histopathology analysis is favorable for fungi detection (Teive et al., 2008; Young et al., 2017) but has no advantage in diagnosing other pathogens. And lastly, the use of RT-PCR is limited by the genetic diversity of the pathogens and the samples size is small due to low pathogens loads. As these conventional methods have limitations in terms of sensitivity, speed, and the spectrum of available assay targets and are unable to satisfy the current needs for fast and precise infection diagnosis, therefore, metagenomic next-Generation sequencing has emerged as needed (Gill et al., 2006; Bragg and Tyson, 2014). Metagenomic next-generation sequencing (mNGS), also known as shotgun deep-sequencing, is a high-throughput sequencing approach that can detect any microorganism in a clinical sample in theory and can distinguish etiologic microorganisms from background commensals with high efficiency and short turnaround time (Miao et al., 2018). As an emerging approach with the ability to detect all potential pathogens in a single assay. the mNGS attracts more and more attention (Miao et al., 2018; Wilson et al., 2019). However, the clinical application of the mNGS remains under discussion in the diagnosis of infections after allo-HSCT. In this study, we compared mNGS and traditional pathogen detection to investigate the pathogens of infections (such as bloodstream infections, pulmonary infections, and CNS infections) in the pediatric allo-HSCT recipients.

## Materials and Methods

### Ethics Statement

This study was carried out according to the principles of the Declaration of Helsinki and approved by the Ethics Review Committee of Guangzhou Women and Children’s Medical Center. Recipients’ approval and informed consent were waived because the study involved a retrospective review of patient records.

### Patients

**FIGURE 1:**
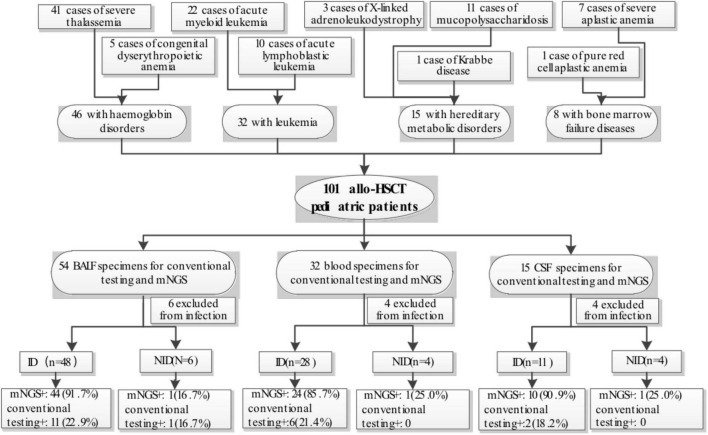
Detailing the characteristic of patient samples.

The study retrospectively reviewed 106 medical records of children with allo-HSCT from Guangzhou Women and Children’s Medical Center between March 2019 and July 2021. These 106 patients have been suspected of bloodstream infections, pulmonary infections, or CNS infections. Five recipients were excluded from the study due to missing critical data. Among the 101 pediatric recipients (34 females and 67 males)enrolled, 46 had hemoglobin disorders (41 cases of severe thalassemia and 5 cases of congenital dyserythropoietic anemia), 32 had leukemia (22 cases of acute myeloid leukemia and 10 cases of acute lymphoblastic leukemia), 15 had hereditary metabolic disorders (11 cases of mucopolysaccharidosis, 3 cases of X-linked adrenoleukodystrophy and 1 case of Krabbe disease) and eight had bone marrow failure disease (7 cases of severe aplastic anemia and 1 case of pure red cell aplastic anemia) (Figure 1). The median age was 6 years old (from 5 months to 14 years old). There were 61 patients within 3 months, 24 patients at 3–6 months, 13 patients at 6–12 months, and 3 patients over more than one year after HSCT. All recipients received infection prophylaxis with acyclovir and posaconazole. Patients and transplantation characteristics were shown in Table 1. The Cytomegalovirus (CMV) and Epstein-Barr virus (EBV) DNA in the peripheral blood were tested by RT-PCR weekly for the first three months and once every 2 weeks from the 4th to the 6th month, then once per month from the 7th to the 12th month after allo-transplantation. Other pathogens were not routinely monitored and were only measured when patients presented with specific symptoms and were suspected of infections.

**TABLE 1:** Patient and transplantation characteristics (*N* = 101).

Patient characteristics	
**SEX, n (%)**	
Male	67 (66.34%)
Female	34 (33.66%)
**Median age at transplant, months**	72 (5∼168)
**Underlying disease, n (%)**	
Severe thalassemia	41 (40.59%)
Acute myeloid leukemia	22 (21.78%)
Acute lymphoblastic leukemia	10 (9.90%)
Mucopolysaccharidosis	11 (10.89%)
Severe aplastic anemia	7 (6.93%)
Congenital dyserythropoietic anemia	5 (4.95%)
X-linked adrenoleukodystrophy	3(2.97%)
Krabbe disease	1(0.99%)
Pure red cell aplastic anemia	1(0.99%)
**Type of donor, n (%)**	
Sibling	17 (16.83%)
Unrelated	50 (49.5%)
Parent	34 (33.66)
**Stem cell source, n (%)**	
Peripheral blood	77 (76.24%)
Cord blood	16 (15.84%)
Marrow + Peripheral blood	8 (7.92%)
**GVHD prophylaxis, n (%)**	
CSA alone	16 (15.84%)
CSA + MTX	17 (16.83%)
CSA + MMF + MTX	59 (58.42%)
PTCy + CSA	9 (8.91%)
**Median time of neutrophil implantation, days after transplantation (+ d)**	
Peripheral blood and Marrow + Peripheral blood	+ 13 (+12∼+16)
UCB	+ 16 (+11∼+28)
**Stage of patient when the specimen was tested, n (%)**	
Within three months after HSCT	61 (60.40%)
At 3–6 months after HSCT	24 (23.76%)
At 6–12 months after HSCT	13 (12.87%)
Over more than one year after HSCT	3 (2.97%)
**Median follow-up time after testing, months**	16 (7∼24)

### Etiological Assessment

Etiological assessment included clinical, imaging, laboratory, and pathogens findings. For patients suspected of pulmonary infection, we completed chest computed tomography (CT) and bronchofibroscopy within 72 h after the onset of symptoms. For patients suspected of having central nervous system infection, CT or/and magnetic resonance imaging (MRI) examination and lumbar punctures were performed within 72 h after symptom onset. The methods for pathogens detection bronchoalveolar lavage fluid (BALF) and cerebrospinal fluid (CSF) included conventional testing and mNGS. For patients suspected of bloodstream infection, infection sites were excluded by imaging examination, and peripheral blood samples were sent for conventional pathogen testing and mNGS. Conventional testing included smear microscopy and culture for bacteria and fungi, RT-PCR for viruses, *Mycoplasma pneumonia*, and *Chlamydia pneumonia*. RT-PCR was performed in accordance with the manufacturer’s protocol, and the assessed viruses included EBV, CMV, adenovirus, human bocavirus, influenza A virus, influenza B virus, parainfluenza virus, respiratory syncytial virus, human metapneumovirus, and enterovirus (Guangzhou HuYanSuo Medical Technology Co., Ltd.) (Liao et al., 2016). mNGS was performed by the BGI PathoGenesis Pharmaceutical Technology, BGI-Shenzhen and Hugobiotech Co., Ltd., Beijing, China according to the manufacturer’s protocol (Miao et al., 2018). According to standard procedures, samples of 0.5–3 mL (BALF, blood, or CSF were collected from patients. Blood samples were stored at room temperature, while all other specimens were stored in liquid nitrogen before testing. The detailed process and positive criteria for mNGS are shown in Supplementary Appendix. The sequencing data of this study can be available at http://ngdc.cncb.ac.cn, reference number PRJCA008385 and https://dataview.ncbi.nlm.nih.gov/object/PRJNA811310.

### Diagnosis of Bloodstream Infections, Pulmonary Infections, and Central Nervous System Infections

The diagnosis of bloodstream infections in accordance with National Healthcare Safety Network (NHSN) criteria was as follows: (1) growth of a recognized pathogen from blood culture, or (2) growth of a commensal organism (e.g., coagulase-negative staphylococci, Micrococcus species) from two blood cultures drawn from different sites at the same time or from the same site at different times on the same or consecutive days (Centers for Disease Control and Prevention [CDC], 2020). For this study, the final diagnosis of bloodstream infections was made based on clinical manifestations, pathogen detection by traditional testing methods and mNGS, and treatment effect observations. In addition to bacteremia and fungal pathogen, all episodes of viremia were included for analysis.

The final diagnosis of pulmonary infection was made based on clinical symptoms, imaging manifestations, pathogen detection by conventional testing methods and NGS, rapid on-site evaluation, histopathologic analysis, expert opinion, and treatment effect observations. Conventional testing methods included smear microscopy and culture for bacteria and fungi, RT-PCR for viruses, *Mycoplasma pneumonia* and *Chlamydia pneumonia*.

The diagnosis of CNS infections was according to the related criteria (Schmidt-Hieber et al., 2016). In brief, confirmed CNS infection was diagnosed as follows: (1) neurological manifestations with normal or abnormal neuroimaging findings; (2) identification of pathogens in CSF; and (3) histopathological evidence if necessary. Probable CNS infection was diagnosed based on neurological manifestations, absence of other non-infectious etiologic evidence or defined diseases, and reasonable response to the antimicrobial treatment.

### Statistical Analysis

Data were statistically analyzed by the SPSS 23.0 software. The *t*-test and χ^2^ test were used for data analysis. Two-by-two contingency tables were derived to determine sensitivity, specificity, positive predictive value (PPV), and negative predictive value (NPV). The measurement data were in accordance with normal distribution, expressed as mean ± standard deviation, and the comparison between groups was performed by *t*-test. Comparative analysis was conducted by Pearson’s χ2 test. *P* values of < 0.05 were considered significant.

## Results

### Sample and Patient Characteristics

In our study, 101 samples Were included for analysis and were categorized into three groups defined as blood samples, BALF samples, and CSF samples (Figure 1). Each group was divided into infectious disease (ID), non-infectious disease (NID). In the blood group (*n* = 32), the majority of patients were diagnosed as ID (28/32, 87.5%), 4 patients were diagnosed as NID. In the BALF group (*n* = 54), 48 (88.9%) patients were diagnosed, six patients were subdivided into NID. in the CSF group (*n* = 15), 11(73.3%) patients Were diagnosed With ID, and four patients Were diagnosed as NID. In the four NID blood patients, two were diagnosed with pre-engraftment syndrome, one was acute graft-vs.-host disease (GVHD), and one was serological reaction caused by antihuman thymocyte globulin (ATG). In the six NID BALF patients, three were transplantation-associated thrombotic microangiopathy (TA-TMA), Two Were bronchiolitis obliterans syndrome, and one was cryptogenic organizing pneumonia. in 4 NID CSF patients, two were subdivided into posterior reversible encephalopathy syndrome, one was TA-TMA, and one was communicating hydrocephalus.

### Pathogens Detected in Bronchoalveolar Lavage Fluid, Blood, and Cerebrospinal Fluid Samples

Among the patients with pulmonary infections, 11 pathogens were identified by both mNGS and conventional testing and 33 was detected by mNGS only. The percentage with the mNGS-positive result was 44/48 (91.7%), included viruses (*n* = 12), bacteria (*n* = 17), fungi (*n* = 9), and mixed infections (*n* = 6). Among the patients diagnosed with fungal pneumonia (*n* = 9), the most prevalent pathogenic fungi were *Pneumocystis jiroveci* (*n* = 6), followed by *Aspergillus* (*n* = 2) and *Mucor circinelloides* (*n* = 1). The most frequently detected viruses were CMV (*n* = 4) and *Human polyomavirus* (*n* = 3). EBV (*n* = 1), adenovirus (ADV, *n* = 1), herpesvirus type 1(HSV-1, *n* = 1), herpesvirus type 4(HSV-4, *n* = 1) and human herpesvirus 6B (HHV-6B, *n* = 1) were also detected. The bacteria detected by mNGS included *Pseudomonas aeruginosa* (*n* = 3), *Streptococcus pneumonia* (*n* = 2), *Klebsiella pneumonia* (*n* = 2), *Staphylococcus haemolyticus* (*n* = 2), *Actinomyces odontolyticus* (*n* = 2), *Acinetobacter baumanii* (*n* = 1), *Enterococcus faecium* (*n* = 1), *Klebsiella aerogenes* (*n* = 1), *Listeria ivanovii* (*n* = 1), *Mycoplasma* (*n* = 1), and *Streptococcus anginosus* (*n* = 1). Patients with mixed infections (*n* = 6) were shown in Table 2; among them, *Pneumocystis jiroveci* (*n* = 4) and *Pseudomonas aeruginosa* (*n* = 3) were the most frequently detected pathogens. The positive results of traditional pathogen detection methods were obtained from 11 (22.9%) patients, the positive pathogens included CMV (*n* = 3), *Pseudomonas aeruginosa* (*n* = 3), *Klebsiella pneumonia* (*n* = 2), *Streptococcus pneumonia* (*n* = 1), *Acinetobacter baumannii* (*n* = 1), and *Pseudomonas maltophilia* (*n* = 1). In patients with pulmonary infections, the pathogens which traditional pathogen detection generally failed to detect included many fungi (such as the *Pneumocystis jiroveci*, *Aspergillus*). In contrast, common bacteria such as *Pseudomonas aeruginosa* and *Klebsiella pneumonia* were relatively able to detect by both traditional methods and mNGS. Due to the limitation of detection reagent range for most viruses, mNGS is more sensitive than traditional pathogen detection tests.

**TABLE 2:** The spectrum of pathogens in pulmonary infections by mNGS (*n* = 48).

Pathogens	Cases
**Virus**	**12**
CMV	4
*Human polyomavirus*	3
ADV	1
EBV	1
HSV-1	1
HSV-4	1
HHV-6B	1
**Bacteria**	**17**
*Pseudomonas aeruginosa*	3
*Streptococcus pneumoniae*	2
*Klebsiella pneumoniae*	2
*Staphylococcus haemolyticus*	2
*Actinomyces odontolyticus*	2
*Acinetobacter baumanii*	1
*Enterococcus faecium*	1
*Klebsiella aerogenes*	1
*Listeria ivanovii*	1
*Mycoplasma*	1
*Streptococcus anginosus*	1
**Fungi**	**9**
*Pneumocystis jiroveci*	6
*Aspergillus*	2
*Mucor circinelloides*	1
**Mixed infections**	**6**
*Pneumocystis jiroveci* + CMV	1
*Pneumocystis jiroveci* + ADV	1
*Pneumocystis jiroveci* + *Pseudomonas aeruginosa*	1
*Pneumocystis jiroveci* + *Acinetobacter baumanii*	1
CMV + *Pseudomonas aeruginosa*	1
*Cunninghamella bertholletiae* + *Pseudomonas aeruginosa*	1
**Unknown**	**4**

*CMV, cytomegalovirus; ADV, adenovirus; EBV, Epstein-Barr virus; HSV-1, Human herpes simplex virus type 1; HSV-4, Human herpes simplex virus type 4; HHV-6B, human herpesvirus 6B.*

Among the 28 blood specimens of patients with bloodstream infections or viremia, five patients were tested positive by both mNGS and conventional methods, nineteen were positive by mNGS, and one was positive by conventional testing only. The percentage with the mNGS-positive result was 24/28 (85.7%), included viruses (*n* = 12), bacteria (*n* = 4), fungi (*n* = 3), and mixed infections (*n* = 5) (Table 3). The most prevalent pathogen was CMV, detected in 11 patients. *Pneumocystis jiroveci* was still the most detected fungus found in three patients. The most commonly detected bacteria were *Pseudomonas maltophilia* (*n* = 3) and *Pseudomonas aeruginosa* (*n* = 2). The positive rate of conventional testing was 6/28 (21.4%), containing *Trichosporon asahii* (*n* = 1), CMV (*n* = 2), and bacteria (*n* = 3, *Pseudomonas maltophilia*, *Pseudomonas aeruginosa*, and *Streptococcus mitis* 1 case each).

**TABLE 3:** The spectrum of pathogens in bloodstream infections or viremia by mNGS (*n* = 28).

Pathogens	Cases
**Virus**	**12**
CMV	6
ADV	3
HHV-6B	2
*Human polyomavirus*	1
**Bacteria**	**4**
*Pseudomonas maltophilia*	2
*Pseudomonas aeruginosa*	1
*Rickettsia felis*	1
**Fungi**	**3**
*Pneumocystis jiroveci*	2
*Trichosporon asahii*	1
**Mixed infections**	**5**
*Pneumocystis jiroveci*+ CMV	1
CMV+ *Pseudomonas aeruginosa*	1
CMV+ *Pseudomonas maltophilia*	1
CMV+ *Cunninghamella blakesleeana*	1
Acinetobacter baumanii + Aspergillus	1
**Unknown**	**4**

*CMV, cytomegalovirus; ADV, adenovirus; HHV-6B, human herpesvirus 6B.*

Of the 15 CSF specimens enrolled, 11 patients were eventually diagnosed with CNS infections. Ten pathogens were detected in 11 patients, including viruses (*n* = 8), bacteria (*n* = 1), and fungi (*n* = 1). Two pathogens were identified by mNGS and conventional testing both and eight by mNGS only. The pathogens detected by both mNGS and conventional testing included CMV (*n* = 1) and *Klebsiella pneumonia* (*n* = 1). The pathogens detected by mNGS only included HHV-7(*n* = 3), HHV-6B (*n* = 2), CMV (*n* = 1), ADV (*n* = 1), and Mucor (*n* = 1). The spectrum of pathogens was shown in Table 4, and viruses were the most frequent causes.

**TABLE 4:** The spectrum of pathogens in CNS infections by mNGS (*n* = 11).

Pathogens	Cases
**Virus**	**8**
HHV-7	3
HHV-6B	2
CMV	2
ADV	1
**Bacteria**	**1**
*Klebsiella pneumoniae*	1
**Fungi**	**1**
**Mucor**	**1**
**Unknown**	**1**

*HHV-7, human herpesvirus-7; HHV-6B, human herpesvirus 6B; CMV, cytomegalovirus; ADV, adenovirus.*

### Sensitivity, Specificity, Positive Predictive Value, and Negative Predictive Value of Metagenomic Next-Generation Sequencing and Conventional Testing

**FIGURE 2:**
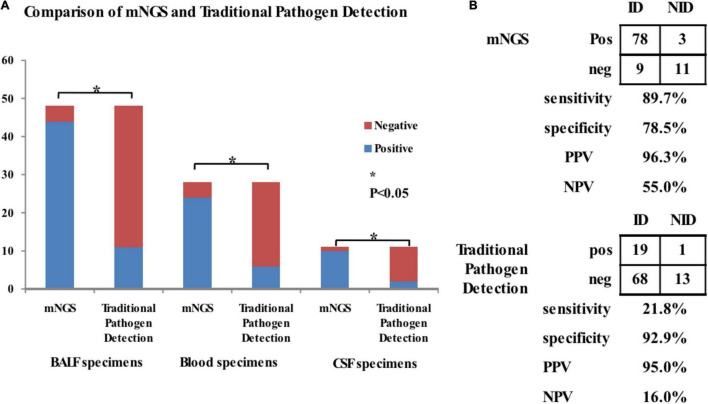
Comparison of positive rates between mNGS and traditional pathogen testing for detection pathogens in BALF (*n* = 48), blood (*n* = 28), and CSF (*n* = 11) samples. **(A)** The sensitivity of mNGS was compared to that of traditional pathogen detection methods for diagnosis of pulmonary infections, bloodstream infections or viremia, and CNS infections in children recipient. The sensitivity of mNGS for diagnosing pulmonary infections, bloodstream infections or viremia, and CNS infections post-transplant were 91.7, 85.7, and 90.9, respectively. In contrast, the sensitivity of conventional testing for diagnosing pulmonary infections, bloodstream infections or viremia, and CNS infections post-transplant were 22.9, 21.4, and 18.2, respectively. There were significant differences in the sensitivity of mNGS and conventional testing in BALF, blood, and CSF samples, with *P*-values of 0.000, 0.000, and 0.002, respectively. **(B)** The sensitivity and specificity of mNGS were compared to traditional pathogen detection methods for diagnosis of infections in post-transplant children. mNGS was more sensitive (89.7%) compared to conventional pathogen detection (21.8%), with a difference of 67.9% (*P* < 0.001), However, mNGS was less specific (78.5%) than traditional methods (92.9%), with a difference of 14.4% (*P* = 0.596). The PPV and NPV of mNGS were 96.3 and 55.0%, respectively. In comparison, the PPV and NPV of traditional pathogen detection were 95.0 and 15.0%, respectively. *P < 0.05.

The sensitivity of mNGS was compared to that of traditional pathogen detection methods (Figure 2A). The sensitivity of mNGS for diagnosing pulmonary infections, bloodstream infections or viremia, and CNS infections post-transplant were 91.7, 85.7, and 90.9%, respectively. In contrast, the sensitivity of conventional testing for diagnosing pulmonary infections, bloodstream infections or viremia, and CNS infections post-transplant were 22.9, 21.4, and 18.2%, respectively. There were significant differences in the sensitivity of mNGS and conventional testing in BALF, blood, and CSF samples, with *P* values of 0.000, 0.000, and 0.002, respectively. However, we did not analyze differences in specificity of each group of specimens because there were few non-infected cases, which would undermine the reliability of the results. Of the 101 patients enrolled, 87 were diagnosed with post-transplant ID and 14 with NID eventually. The sensitivity and specificity of mNGS were compared to traditional pathogen detection methods (Figure 2B). mNGS was more sensitive (89.7%) compared to conventional pathogen detection (21.8%), with a difference of 67.9% (*P* < 0.001), However, mNGS was less specific (78.5%) than traditional methods (92.9%), with a difference of 14.4% (*P* = 0.596). The PPV and NPV of mNGS were 96.3 and 55.0%, respectively. In comparison, the PPV and NPV of traditional pathogen detection were 95.0 and 15.0%, respectively.

### Treatment and Outcome

A total of 87 patients with post-transplant infections, including 48 pulmonary infections, 28 bloodstream infections or viremia, and 11 CNS infections, received treatment. Treatments were given based on the pathogens, and empirical treatments were provided for those with unclear pathogens. Of the patients with pulmonary infections, 40 had a good response to the treatments and survived, While four died of TMA and two died of aGVHD although with improving pulmonary infections on the third day of treatment, and two died because of pulmonary infections progressing. Twenty-seven of the patients with bloodstream infections or viremia responded well during the treatments, while one died of multiple organ failure. Ten of the patients with CNS infections had a good response to the treatments, while one died because of CNS infections progressing (the pathogen of the patient was Mucor) and multiple organ failure.

## Discussion

Our study retrospectively evaluated the application of mNGS in pathogen detection after allo-HSCT in children by performing mNGS and traditional pathogen detection in specimens obtained from blood, CSF, and BALF. All of the patients enrolled in this study had immune deficiency due to the use of large doses of anti-tumor drugs, cytotoxic drugs, immunosuppressants and glucocorticoids associated with hematopoietic stem cell transplantation. These patients were more prone to infection, so rapid and accurate identification of pathogens and timely use of targeted anti-infective drugs were extremely important. The mNGS analysis provides great potential for pathogens identification, by which nearly all potential pathogens can be accurately identified in a single method without the need for sequence-specific amplification. mNGS is widely used in clinical practice. Miao et al. indicated that mNGS had a higher sensitivity (52.5 vs. 34.2%) and a similar specificity (85.7 vs. 89.1%) compared with traditional pathogen detection methods among immunocompetent individuals with acute or chronic infections (Miao et al., 2018). And Huang et al. reported similar results among patients with a deficient or normal immune function who developed pulmonary infections (Huang et al., 2020). The primary objective of this study was to evaluate the clinical usefulness of mNGS in identifying the pathogens of infections in a setting of patients after allo-HSCT. Liu et al. showed that the sensitivity of mNGS and conventional testing in the diagnosis of adult CNS infections post-HSCT were 97.1 and 82.9%, while the specificity of mNGS and conventional testing were 94.4 and 100% (Liu et al., 2021). However, data on mNGS in post-transplant infections in children are lacking. Our results showed that the sensitivity of mNGS (89.7%) was significantly higher than conventional testing (21.8%), while the specificity of mNGS (78.5%) was less than conventional testing (92.9%) but not significant among children infections post-allo-HSCT. mNGS seemed to have a trend of higher sensitivity and a similar specificity to identify the pathogens of infections in allo-HSCT children compared with conventional testing. We also compared the sensitivity of mNGS to conventional testing for the diagnosis of post-transplant infections in different specimens (including BALF, blood, and CSF). The sensitivity of mNGS for diagnosing pulmonary infections, bloodstream infections or viremia, and CNS infections post-transplant in children was apparently higher than that of conventional testing. Of interest, the sensitivity of mNGS in our study was similar to those in some previously reported results (Liu et al., 2019; Huang et al., 2020), but higher than those in some other studies (Miao et al., 2018; Miller et al., 2019). These discrepancies might be due to the different enrolled cohorts, reference standards, as well as the different types of infectious diseases. The patients enrolled in this study were infected after allo-HSCT, with immune deficiency, and were particularly prone to virus and fungal infections in which mNGS was considered sensitive (Liu et al., 2019; Huang et al., 2020). In addition, the reference standard was based on the clinical diagnosis rather than the results of routine testing, which might be inaccurate in some cases (Liu et al., 2019; Huang et al., 2020). Large sample-size may be needed to test the difference.

Metagenomic next-generation sequencing technology has been widely used in clinical practice to diagnose infectious diseases. Increasing evidence (Lavezzo et al., 2016; Deurenberg et al., 2017) has demonstrated the superiority of mNGS in diagnosing unknown etiology, particularly regarding the detection of viruses and antibiotic-resistant bacteria. Huang et al. (2020) showed that mNGS has advantages for pathogen detection in cases of mixed pulmonary infections in immune-impaired patients and is more effective for fungus than for bacteria identification. In our study, mNGS detected 87 children infected after hematopoietic stem cell transplantation, and the total positive rate was 89.7, among which 36.8% were viruses, 25.3% were bacteria, 14.9% were fungi, and 12.6% were mixed infections. The sensitivity of mNGS for detecting respiratory pathogens (human metapneumovirus, respiratory syncytial virus, human herpesvirus 6, CMV, and *Stenotrophomonas maltophilia*) in BALF from HSCT patients with acute respiratory illnesses was reported to be 100%, which is higher than the rate tested by conventional methods (Langelier et al., 2018). In 13 cases of pneumocystis pneumonia, P. jiroveci was detected in all the BALF, sputum, and blood samples by mNGS and in 5/13 samples by conventional testing, demonstrating the superior sensitivity of the former method (Zhang et al., 2019). Wang et al. (2020) showed that mNGS was especially useful for detecting fungi. The fungal pneumonia was identified in only 1/21 of samples by the culture method but 19/21 by mNGS (Langelier et al., 2018; Wang et al., 2020). This is consistent with our findings. In our study, *Pneumocystis Jiroveci* was the most frequent pathogen in the BALF, which were detected in 10 of 48 patients with pulmonary infections post-transplantation, among which four cases were mixed infections. While in CNS infections post-transplantation, viruses, especially herpesviruses were most one detected (Wu et al., 2013; Abidi et al., 2019). However, some retrospective studies revealed that HHV-6 was the most common one (Hanajiri et al., 2017; Abidi et al., 2019). A prospective study from China suggested that EBV was the most common cause of CNS viral infections in HSCT recipients (Liu et al., 2019). Viruses (9/11) were found to be the leading cause of CNS infections in the current study. In our study, CNS infection samples are few, further study is needed to confirm these findings.

The mNGS results involved the process of specimen collection, removing host sequences, and identification of pathogen species (pathogenic, contaminating or colonizing bacteria). In our study, nine mNGS results are false negative and three are false positive. Firstly, one possible reason is all patients with suspected infection were included in the study, but the final diagnosis of infection still required strict screening, including clinical symptoms and signs, imaging features, etiological results, prognosis, and exclusion of non-infectious diseases. Secondly, our mNGS tests were delivered to the centralized laboratory rather than an in-house microbiology laboratory, which may sacrifice sensitivity rate because of reduced viability due to increased turnaround time from bedside to bench. Moreover, the possible reason may be associated with the sample types included. BALF were obtained from the respiratory tract, which is typically contaminated with oral normal flora, commensal organisms, and colonizers, leading to a relatively lower purity than other sample types. Finally, our mNGS is still not truly comprehensive. RNA-Seq data, for example, were not concomitantly tested with DNA sequencing, which might provide valuable complementary information such as RNA virus and microbial transcriptome alterations. However, mNGS detection takes less time and is relatively sensitive, which can provide a good guide for the clinical application of antibiotics (Langelier et al., 2018; Liu et al., 2019; Wang et al., 2020). In our total of 87 patients with post-transplant infections, the vast majority of mNGS positive patients were consistent with our initial clinical judgment. For some patients with unclear initial diagnosis, sensitive antibiotics were adjusted in time after the mNGS positive results were obtained, resulting in a good overall prognosis. There was only three patients died due to infection progression. Due to the use of chemoradiotherapy drugs, immunosuppressants, and the possible presence of GVHD, the patient’s immune function was extremely low after transplantation. Rapid and effective pathogen detection is very important for clinical judgment, and mNGS is a recommended method at present.

In the meantime, there is some limitations for this study. Firstly, we haven’t compare the specificity of mNGS between different types of samples. This is because there were few negative cases, making the calculated specificity unreliable. Secondly, the sample processing procedures used in this study underestimated the possibility of RNA virus infection. If RNA virus infection is suspected, RNA extraction and reverse transcription procedures should be applied. Thirdly, samples was achieved from a single-center retrospective study, more prospective and multicenter data may help to determine the accuracy of mNGS analysis.

## Conclusion

In conclusion, mNGS might be a promising way for the diagnosis of infections post HSCT in children. *Pneumocystis jiroveci* was the most frequent pathogen of pulmonary infections post-transplantation, while viruses were the most common pathogens for the CNS infections in allo-HSCT recipients. More prospective and multicenter data are required to accurately determine the accuracy of mNGS analysis and distribution of pathogens.

## Data Availability Statement

The datasets presented in this study can be found in online repositories. The names of the repository/repositories and accession number(s) can be found below: https://ngdc.cncb.ac.cn/bioproject/browse/PRJCA008385; https://www.ncbi.nlm.nih.gov/bioproject/PRJNA811310.

## Ethics Statement

The studies involving human participants were reviewed and approved by Medical Ethics Committee of Guangzhou Women and Children Medical Center. Written informed consent to participate in this study was provided by the participants’ legal guardian/next of kin.

## Author Contributions

YQ and HJ: conceptualization and funding acquisition. YQ and WD: data curation and resources. YQ, WD, SL, and XW: formal analysis. YQ: investigation and writing—original draft. YQ and HL: methodology. XW, PW, HX, and YC: software. HJ: supervision and writing—review and editing. All authors read and approved the final manuscript.

## Conflict of Interest

HX was employed by Hugobiotech Co., Ltd. YC was employed by BGI PathoGenesis Pharmaceutical Technology Co., Ltd. The remaining authors declare that the research was conducted in the absence of any commercial or financial relationships that could be construed as a potential conflict of interest.

## Publisher’s Note

All claims expressed in this article are solely those of the authors and do not necessarily represent those of their affiliated organizations, or those of the publisher, the editors and the reviewers. Any product that may be evaluated in this article, or claim that may be made by its manufacturer, is not guaranteed or endorsed by the publisher.
