# Supplementary material for: Metagenomic Next-Generation Sequencing vs. Traditional Pathogen Detection in the Diagnosis of Infection After Allogeneic Hematopoietic Stem Cell Transplantation in Children
Source: Front Microbiol. 2022 Apr 18;13:868160. doi: 10.3389/fmicb.2022.868160 (PMC9058167; doi:10.3389/fmicb.2022.868160)
Supplement: Supplementary file 1 [file Presentation_1.pdf]

Detailed Process and Positive Criteria for mNGS

Detailed Process

Blood samples were stored at room temperature, while all other specimens were stored in liquid nitrogen before testing. Volumes of 3–4 mL of blood were drawn from patients, placed in ethylenediaminetetraacetic acid tubes, stored at room temperature for 3–5 minutes before plasma separation and centrifuged at 1600*g* for 10 minutes at 4°C within 8 hours of collection. Plasma samples were transferred to new sterile tubes. Samples of 0.5–3 mL Cerebrospinal fluid（CSF） or bronchoalveolar lavage fluid (BALF) were collected from patients according to standard procedures. Then, 1.5-mL microcentrifuge tubes with 0.5 mL of sample and 1 g of 0.5-mm glass beads were attached to a horizontal platform on a vortex mixer and agitated vigorously at 2800–3200 rpm for 30 min. After agitation, 0.3 mL of the sample was separated into a new 1.5-mL microcentrifuge tube, and DNA was extracted using a TIANamp Micro DNA Kit (DP316, Tiangen Biotech) according to the manufacturer's recommendation.The extracted DNA samples were used to construct a DNA library based on the Beijing Genomics Institute sequencer-100; the library construction process included DNA end repairing, adapter connection, polymerase chain reaction (PCR) amplification, and amplification product purification by magnetic beads (MGI, Shenzhen, China). The Agilent 2100 Bioanalyzer (Agilent Technologies, Santa Clara, Canada) and quantitative polymerase chain reaction (qPCR) were used for library quality control. The quality control qualified DNA library was sequenced on the Beijing Genomics Institute sequencer-100 platform (BGI, Shenzhen, China and Hugobiotech Co. Ltd, Beijing, China).

High-quality sequencing data were generated by removing low-quality and short (length <35 bp) reads, followed by computational subtraction of human host sequences mapped to the human reference genome (hg19) using Burrows-Wheeler alignment. The data remaining after removal of low-complexity reads were classified by simultaneous alignment to 4 microbial genome databases consisting of viruses,

bacteria, fungi, and parasites. The classification reference databases were downloaded from National Center Biotechnology Information (ftp://ftp.ncbi.nlm.nih.gov/genomes/). RefSeq contains 4189 whole-genome sequences of viral taxa, 2328 bacterial genomes or scaffolds, 199 fungi related to human infection, and 135 parasites associated with human diseases.

Criteria for a Positive mNGS Result

1. Virus, bacteria (mycobacteria excluded), and parasites:according to Langelier's study, mNGS identified microbes as confirmed pathogens following the microbes which were identified by the clinical test. Microbes were considered as potential pathogens if literature has reported the score orthe pathogenicity were as least tentimes greater than that of any other microbes of the same type identified in the patient[1].

2. Fungi: mNGS identified microbes (species level) as confirmed pathogens when thecoverage rate scored 5 times higher than that of any other fungus,since low absolute abundance appears in contaminated fungal DNA and many samples[2, 3].

3. Mycobacteria: Mycobacterium tuberculosis (MTB) was identifiedpositive when at least 1 read mappdto species or genus level,since the Mycobacteria cell wall ishard to disrupt the organism to release nucleic acid as well as low possibility for contamination [4, 5]. The efficient methods of extraction are crucialto achieve unbiased sequencing. Nontuberculous mycobacteria (NTM) were considered aspositive when the number of mapped reads (genus or specieslevel) was top 10 in the bacteria list due to the low yield rate and balanceof environmental contamination from hospitaltolaboratory [6, 7].

1. Langelier, C., et al., Metagenomic Sequencing Detects Respiratory Pathogens in Hematopoietic Cellular Transplant Patients. Am J Respir Crit Care Med, 2018. 197(4): p. 524-528.

2. Bittinger, K., et al., Improved characterization of medically relevant fungi in the human respiratory tract using next-generation sequencing. Genome Biol, 2014. 15(10): p. 487.

3. Schlaberg, R., et al., Validation of Metagenomic Next-Generation Sequencing Tests for Universal Pathogen Detection. Arch Pathol Lab Med, 2017. 141(6): p. 776-786.
4. Doughty, E.L., et al., Culture-independent detection and characterisation of Mycobacterium tuberculosis and M. africanum in sputum samples using shotgun metagenomics on a benchtop sequencer. PeerJ, 2014. 2: p. e585.
5. Simner, P.J., S. Miller, and K.C. Carroll, Understanding the Promises and Hurdles of Metagenomic Next-Generation Sequencing as a Diagnostic Tool for Infectious Diseases. Clin Infect Dis, 2018. 66(5): p. 778-788.
6. Ozcolpan, O.O., et al., [Distribution of nontuberculous mycobacteria isolated from clinical specimens and identified with DNA sequence analysis]. Mikrobiyol Bul, 2015. 49(4): p. 484-93.
7. van Ingen, J., et al., Global outbreak of severe Mycobacterium chimaera disease after cardiac surgery: a molecular epidemiological study. Lancet Infect Dis, 2017. 17(10): p. 1033-1041.
